# Utility of magnetic resonance imaging in the evaluation of left ventricular thickening

**DOI:** 10.1007/s13244-017-0549-2

**Published:** 2017-03-09

**Authors:** Nicholas Fulton, Prabhakar Rajiah

**Affiliations:** 1 Department of Radiology, University Hospital Case Medical Center, Cleveland, OH USA; 2 Department of Radiology Cardiothoracic Imaging, UT Southwestern Medical Center, E6.120 B, Mail code 9316, 5323 Harry Hines Boulevard, Dallas, TX 75390-8896 USA

**Keywords:** Cardiac, MRI, LV thickening, Hypertension, Hypertrophy

## Abstract

**Abstract:**

Left ventricular (LV) thickening can be due to hypertrophy (concentric, asymmetric, eccentric) or remodelling (concentric or asymmetric). Pathological thickening may be caused by pressure overload, volume overload, infiltrative disorders, hypertrophic cardiomyopathy, athlete’s heart or neoplastic infiltration. Magnetic resonance imaging (MRI) plays an important role in the comprehensive evaluation of LV thickening, including: establishing diagnosis, determining LV geometry, establishing aetiology, quantification, identifying prognostic factors, serial follow-up and treatment response. In this article, we review the aetiologies and pathophysiology of LV thickening, and demonstrate the comprehensive role of MRI in the evaluation of LV thickening.

***Teaching Points*:**

• *MRI plays an important role in the evaluation of LV thickening*.

• *LV thickening can be due to either hypertrophy or remodelling*.

• *Pathological thickening can be due to pressure/volume overload or infiltrative disorders*.

**Electronic supplementary material:**

The online version of this article (doi:10.1007/s13244-017-0549-2) contains supplementary material, which is available to authorized users.

## Introduction

Left ventricular (LV) thickening is a commonly encountered finding in several imaging modalities. LV thickening can be either due to hypertrophy or remodelling. Pathological LV thickening may be caused by pressure/volume overload, infiltrative disorders, hypertrophic cardiomyopathy, athlete’s heart or neoplastic infiltration (Table 1) [1]. LV hypertrophy (LVH) is characterized by increased length and width of the myocyte, as opposed to hyperplasia, which is characterized by generation of new myocytes. The development of hypertrophy is a complex process that involves myocardial stress, growth factors, cytokines, catecholamines and genetic abnormalities [2]. As per Laplace’s law, the load in the myocardium is (pressure x radius)/ (2 x wall thickness). In pressure overload, hypertrophy is caused by increased myosin heavy chain synthesis, whereas in volume overload, hypertrophy is caused by decreased myosin heavy chain degradation. Both these overloads also result in reprogramming of several genes, (e.g., ANP, ACE, SERCA-2, BBR, M2, etc.), which activate matrix metalloproteinase (MMPs) and increase the synthesis of collagen 1 resulting in fibrosis [3].

**Table 1:** Causes of LV thickening

Pressure overload	Hypertension Aortic stenosis Coarctation Subaortic membrane
Volume overload	Aortic/mitral regurgitation Dilated cardiomyopathy
Infiltrative cardiomyopathies	Amyloidosis Anderson-Fabry disease Danon disease Eosinophilic heart disease Mucopolysaccharidoses Friedreich’s ataxia Myocardial oxalosis Pompe disease Sarcoidosis Iron overload
Non infiltrative cardiomyopathy	Hypertrophic cardiomyopathy
Adaptive response	Athlete’s heart
Miscellaneous	Neoplastic infiltration

Regardless of the aetiology, LV thickening is an adverse prognostic determinant and requires treatment [1]. LV thickening is associated with higher risk of cardiovascular morbidity and mortality, independent of other traditional risk factors [4–6]. There is variability in these outcomes depending on the aetiology, LV geometry and ethnicity [1]. Regression of LVH has been shown to be associated with decreased risk and improved outcomes [7]. Imaging plays a vital role in the diagnosis and management of LV thickening. In this article, we review the comprehensive role of MRI in the evaluation of LV thickening and discuss the utility of MRI in several aetiologies of LV thickening.

## Imaging of left ventricular thickening

Several imaging modalities are available and utilized in the evaluation of LV thickening, including echocardiography, computed tomography (CT) and MRI, each with their own advantages and disadvantages.

### Echocardiography

Echocardiography is the most commonly used imaging modality in the evaluation of LV thickening. LV thickness and mass is reliably measured using the M-mode [8]. Systolic and diastolic functions are quantified using real time cine imaging. Although 2D echocardiography has been shown to be less accurate and reliable than MRI in the evaluation of the LV mass [9], 3D echocardiography has good accuracy and reproducibility [1]. Doppler technique is used to evaluate valvular and vascular lesions. Limitations of echocardiography include poor acoustic windows, attenuation and operator dependence [1].

#### Computed tomography

CT is also occasionally used to evaluate LV thickening, particularly in patients with contraindications for MRI. CT can outperform conventional echocardiography in diagnosing and quantifying the amount of LVH [10]. However, CT has been shown to overestimate the LV volume [11] and the reliability of ejection fraction is variable [12]. CT is also associated with ionizing radiation, potentially nephrotoxic contrast media and lower temporal resolution [13].

## MRI

MRI is the most valuable imaging modality in the evaluation of LV thickening and enables comprehensive evaluation of LV thickening, including establishing diagnosis of LV thickening, determining LV geometry, establishing aetiology, quantification, identifying prognostic factors, serial follow-up and assessment of treatment response. In certain instances, particularly with suboptimal echocardiography, MRI is used in establishing the diagnosis of LV thickening. MRI can exquisitely demonstrate the specific LV geometric pattern, which has therapeutic and prognostic implications (see below). The tissue characterization capabilities of MRI help in establishing a specific aetiology of LV thickening, which is essential for determining the appropriate treatment strategy. MRI is considered the gold standard in quantification of LV mass as well as volumes and function due to its high accuracy and reproducibility [1]. This makes MRI ideal for serial measurements of LV mass in clinical scenarios as well as research trials to assess treatment response. MRI provides prognostic information based on mass, volumes, function, and fibrosis. MRI can evaluate other structures, including vasculature and valves [14]. Several MRI sequences are used in the evaluation of LV thickening. ECG-gated steady-state free precession (SSFP) cine sequence is used in morphological evaluation of the LV thickening as well as qualitative evaluation of ventricular function. LV mass, volumes and function can also be quantified from a stack of short axis slices by drawing endocardial and epicardial contours, both in the end-diastolic and end-systolic phases. Regional myocardial function can be accurately evaluated using several deformation techniques such as feature tracking, myocardial tagging, strain-encoded MRI (SENC) and displacement encoding with stimulated echoes (DENSE) [15]. Some of these techniques such as myocardial tagging require dedicated post processing software and additional time, which has precluded widespread clinical adoption of such techniques. Velocity-encoded phase-contrast (PC) sequence is used in the quantification of valvular lesions (stenosis, regurgitation), other obstructive lesions (coarctation) and shunts [16]. T2-weighted sequences (STIR, SSFP) are used in the evaluation of myocardial oedema. Post-contrast dynamic first pass perfusion imaging can identify perfusion defects (e.g., aortic stenosis, hypertrophic cardiomyopathy) [17]. Late gadolinium enhancement (LGE) sequences are used to detect scar/fibrosis, which helps in establishing aetiology of LV thickening as well as determining prognosis. T1 mapping sequences, both pre and post contrast, quantify the T1 values as well as extracellular volume (ECV), which is more sensitive in the detection of diffuse fibrosis than LGE. Additionally, T1 mapping can identify deposits of amyloid, lipid and iron [18–20]. MR angiography (MRA) is useful in the evaluation of vascular abnormalities such as coarctation and renal arterial stenosis. The 4D flow sequences are useful in the assessment of flow characteristics such as direction and vortices, which may have a potential future clinical role.

## MRI parameters in LV thickening

**Fig. 1:**
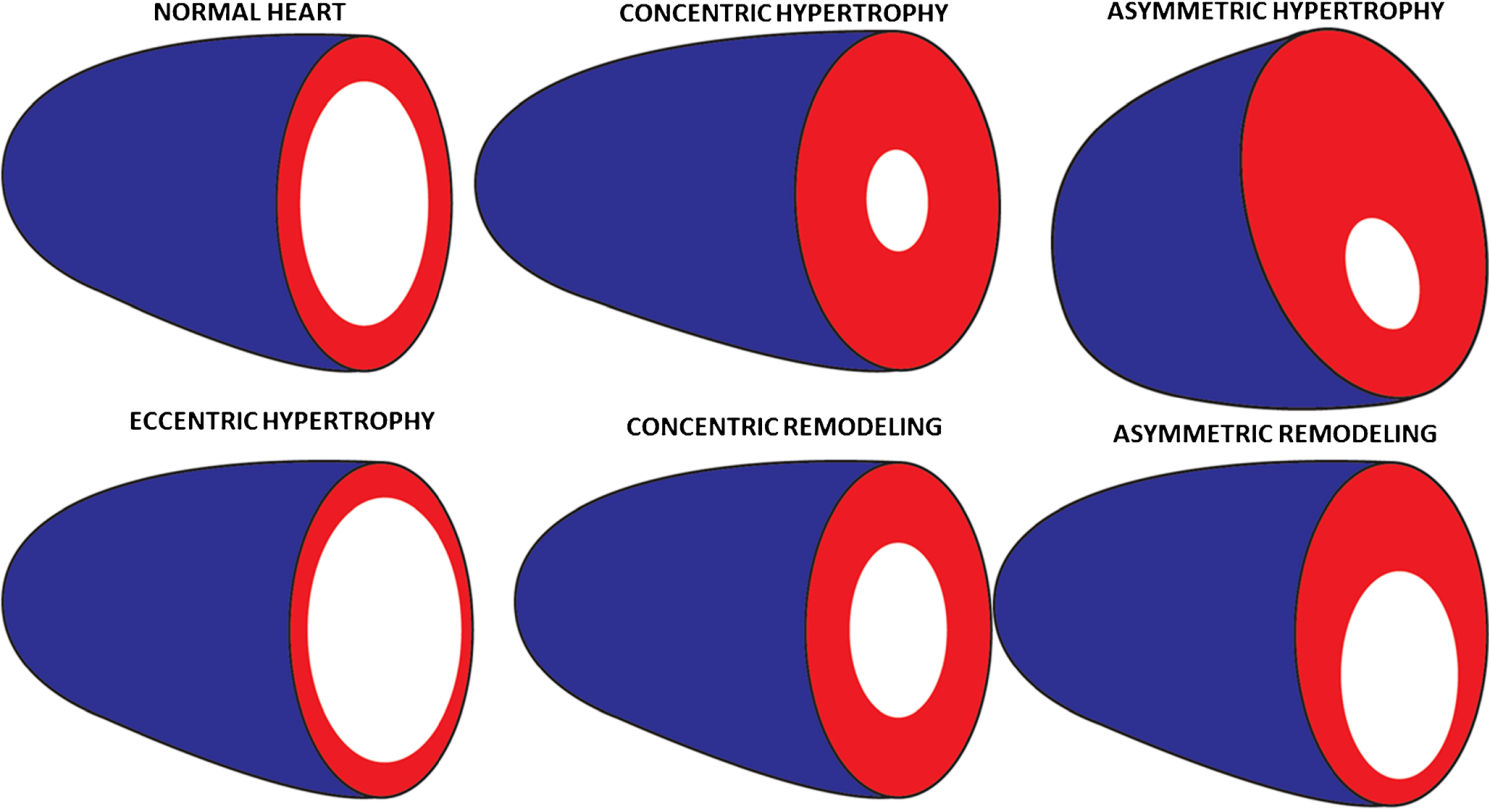
Illustration showing the various types of left ventricular thickening. In a normal heart, the LV wall thickness, septal/lateral wall ratio, LV mass, LV volume, relative wall mass and ejection fraction are normal; in concentric hypertrophy, the wall thickness, LV mass and relative wall mass are increased. While septal/lateral wall ratio is normal, the LV volume is normal or increased and ejection fraction is normal or decreased; in asymmetric hypertrophy, the wall thickness, LV mass and relative wall mass are increased, but the septal/lateral wall ratio is increased. In eccentric hypertrophy, the wall thickness and septal/lateral wall ratio are normal, but LV mass and LV volume are increased, while ejection fraction and relative wall mass are decreased. In concentric remodelling, the relative wall mass is increased, the LV volume is decreased, but septal/lateral wall ratio, wall thickness, LV mass and ejection fraction are normal. In asymmetric remodelling, the relative wall mass is increased and LV volume is decreased. While septal/lateral wall ratio is increased, wall thickness, LV mass and EF are normal

On MRI, several measurements are utilized to quantify and characterize LV thickening. **LV wall-thickness (LVWT)** measured in an end-diastolic cine image is the most commonly used measurement. Normal LV myocardial thickness is < 11 mm. Myocardial thickening is considered mild if it measures 11–13 mm, moderate if it measures 14–15 mm and severe if it measures > 15 mm. **LV mass (LVM**) is quantified from end-diastolic phase images of short axis cine SSFP stack by drawing endocardial and epicardial contours. Using the modified Simpson’s rule, the myocardial volume is obtained and multiplying this by myocardial density (1.05 g/ml) gives the myocardial mass. An absolute mass > 184 g in males and 144 g in females, BMI-indexed mass > 91 g/m2 in males and > 77 g/m2 in females and height-indexed mass of > 83 g/m in males and > 69 g/m in females is considered LV thickening [1, 21, 22]. The height indexed mass is more reliable in obese patients, and also has been shown to correlate better with adverse cardiovascular events and mortality [23]. Other parameters that are crucial in classifying LV thickening are – **Relative wall mass (RWM)**, which is the ratio of LVM/LVEDV (end-diastolic volume), normally < 1.16 (Fig. 1); and **Septal/lateral wall thickness**, which is the ratio of thickness of septal and lateral segments. This ratio is normally 1, but is abnormal in patients with asymmetric hypertrophy or remodelling.

## Geometrical patterns of LV thickening

There are five different geometric patterns of LV thickening that can be exquisitely demonstrated using MRI, namely concentric hypertrophy, asymmetric hypertrophy, eccentric hypertrophy, concentric remodelling and asymmetric remodelling (Table 2). Broadly, the hypertrophic geometries are associated with increased LWM, while the remodelling geometries are associated with normal LWM (Fig. 1). **Concentric hypertrophy** is characterized by increased LVWT, LVWM and RWM, but with normal septal/lateral wall ratio. LV volume is normal or increased and EF is normal or decreased. This pattern is seen in pressure overload as well as infiltrative cardiomyopathies. **Asymmetric hypertrophy** is characterized by increased LVWT, LVWM, and RWM, but with an increased septal/lateral wall ratio. LV volume can be normal or decreased and EF can be normal or increased. **Eccentric hypertrophy** is characterized by increased LVWT and LVWM, but the RWM is subnormal (<1.16) due to increased LV volume and the septal/lateral ratio is normal. EF is normal or decreased. Athlete’s heart is classic example of eccentric hypertrophy presenting with a normal EF. **Concentric remodelling** is characterized by normal LVWT and LVWM, but the RWM is high due to low LV volume. Septal/lateral ratio and EF are normal. **Asymmetric remodelling** is characterized by normal LVWT and LVWM, but the RWM is high and EF is normal. Unlike concentric remodelling the septal/lateral ratio is elevated. Concentric hypertrophy, eccentric hypertrophy and concentric remodelling are all associated with adverse events, with the highest risk seen in concentric hypertrophy [24].

**Table 2:** Types of LV thickening and their distinguishing characteristics

	Wall thickness	Septal/lateral wall ratio	LV mass	LV volume	Relative wall mass (LVM/EDV)	Ejection fraction
Normal	Normal	Normal	Normal	Normal	Normal	Normal
Concentric remodeling	Normal	Normal	Normal	Decreased	Increased	Normal
Asymmetric remodeling	Normal	Increased	Normal	Decreased	Increased	Normal
Concentric Hypertrophy	Increased	Normal	Increased	Normal/Increased	Increased	Normal/decreased
Eccentric hypertrophy	Normal	Normal	Increased	Increased	Normal/subnormal	Decreased
Asymmetric hypertrophy	Increased	Increased	Increased	Normal/decreased	Increased	Normal/increased
Infiltrative disease	Increased	Normal	Increased	Normal/decreased	Increased	Decreased/normal

## Etiologies of LV thickening

**Fig. 2:**
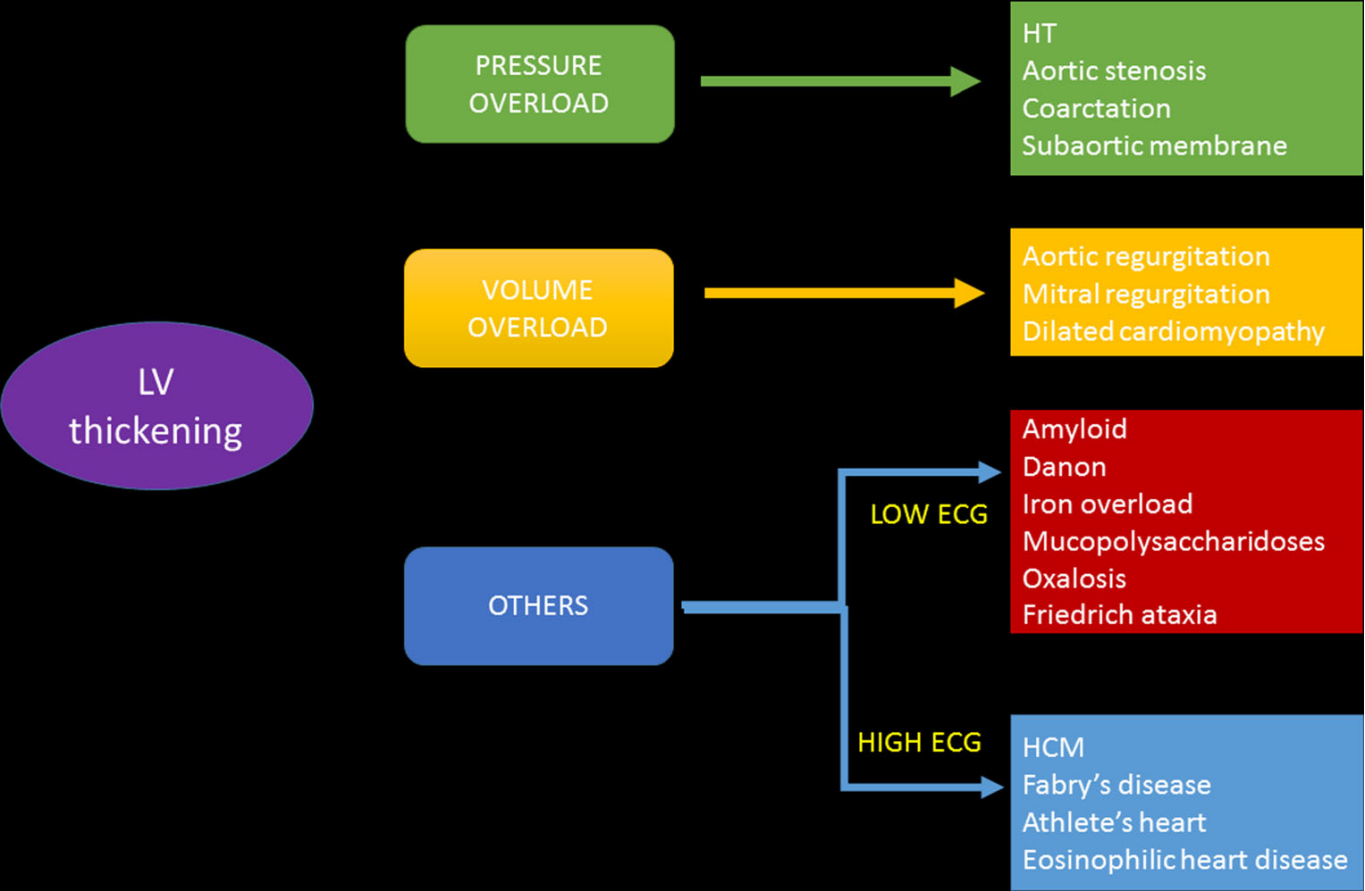
Flow chart showing the differentiation of various causes of LV thickening

The several aetiologies of LV thickening are discussed in Table 2. A flow chart to distinguish these entities is shown in Fig. 2. The comprehensive role of MRI in the evaluation of LV thickening is summarized in Table 3. The most common aetiologies are discussed in detail below.

**Table 3:** Summary of the role of MRI in LV thickening

Disease	Role of MRI
General	Establishing the presence of hypertrophy Characterizing the geometry Quantification of volumes, masses and function Establishing aetiology Fibrosis- Prognostic determinant Evaluating regression
Systemic hypertension	Secondary causes of hypertension- renovascular, adrenal lesion
Aortic stenosis	Planimetry- Aortic valve area Phase contrast- Quantification of stenosis Fibrosis Evaluation for TAVI
Coarctation	Identification of coarctation/hypoplasia Quantification of narrowing Collaterals Associated abnormalities
Subaortic membrane	Membrane- Location, size Quantification of obstruction
Aortic/mitral regurgitation	Quantification of regurgitation Volume quantification
Dilated cardiomyopathy	Quantification of volumes Fibrosis
Amyloidosis	T1 kinetics and T1 mapping- Diagnosis and prognosis Late gadolinium enhancement
Danon disease	Fibrosis
Hypertrophic cardiomyopathy	Pattern of HCM Quantification of obstruction Mitral valve abnormalities- SAM, elongated anterior leaflet Papillary muscle abnormalities Fibrosis
Athlete’s heart	Quantification of volumes Response to deconditioning
Anderson Fabry disease	T1 mapping allows detection of fibrosis and lipid deposition
Iron overload cardiomyopathy	Myocardial iron quantification

## Pressure overload

### HT

**Fig. 3:**
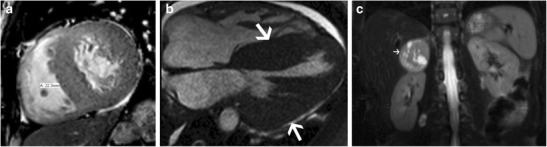
**a** Short axis cine-SSFP image in a patient with hypertension shows severe concentric LV hypertrophy with maximal thickness of 2.2 cm in the mid septum in end diastole. **b** 4-chamber cine SSFP image in another patient with HT shows severe concentric LV hypertrophy. **c** Coronal T1-weighted image through the upper abdomen after gadolinium administration shows an intensely enhancing mass in the upper abdomen (arrow), consistent with a pheochromocytoma

Systemic hypertension (HT) can either be primary or secondary to lesions such as renal artery stenosis, adrenal tumours or coarctation. It has a prevalence of 20–30% [1] with LVH seen in 30% of HT patients. LVH in HT is predominantly of a concentric hypertrophic pattern (Fig. 3a, b) [1] and HT is the most common cause of concentric LVH. Eccentric hypertrophy, concentric remodelling or a normal morphology can also be seen in HT [25]. The EF is preserved, and there is no systolic anterior motion of mitral valve (SAM) or LVOT obstruction. Left atrial enlargement and diastolic dysfunction may be present, both of which have prognostic value [1]. LV dilation and systolic dysfunction may be seen in advanced cases with ventricular failure. Myocardial tagging shows depressed circumferential strain, most prominently in the septum [1]. No perfusion defect is identified per se, but may be seen due to associated coronary artery disease. LGE may be seen in 20-45% due to supply–demand mismatch, coronary microangiopathy or diffuse interstitial fibrosis [1, 26]. LGE is seen in a mid-myocardial distribution either in the basal septal or inferolateral segments [1]. T1- mapping shows diffuse fibrosis, more common in eccentric LVH and is associated with systolic impairment, whereas concentric remodelling is associated only with abnormal aortic function [25]. Vascular changes of HT seen in MRI include atheroma, ulcer, intramural hematoma, dissection and occlusive disease. These can be visualized in sequences such as high-resolution black-blood T1 and T2-weighted fast spin-echo as well as contrast-enhanced 3d MRA sequences. MRI is valuable in establishing the aetiology of secondary hypertension, such as renal arterial stenosis/coarctation with MR angiography and adrenal adenoma/pheochromocytoma (Fig. 3c) with multi-sequence MRI. MRI can be used to follow up response to therapy with decrease of LVH.

A diagnostic dilemma is to distinguish concentric hypertrophy due to HT from a concentric type of hypertrophic cardiomyopathy (HCM). On MRI, HT patients have normal or reduced EF, increased chamber volumes and occasional fibrosis, while HCM patients have normal or increased EF, normal chamber volumes and higher incidence of focal/diffuse fibrosis. Although not widely available, strain imaging can help in distinguishing these entities, since HT patients have high wall stress and lower anteroseptal systolic strains, while HCM patients have decreased wall stress and longitudinal strain [2].

### Aortic stenosis

**Fig. 4:**
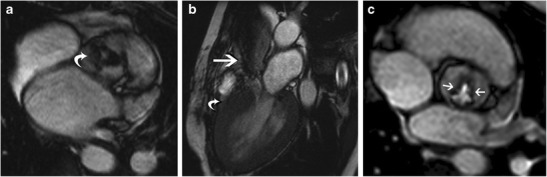
**a** Short axis cine SSFP image through the aortic valve shows thickening of the valve leaflets (arrow) and restricted systolic opening. The valve area was measured to be 0.5 cm2, consistent with severe aortic stenosis. **b** 3-chamber cine SSFP sequence in the same patient shows restricted systolic opening of the aortic valve and flow acceleration through the aortic valve (straight arrow), consistent with aortic stenosis. Concentric LV hypertrophy is also seen (curved arrow). **c** Short axis cine SSFP image through the aortic valve shows a unicuspid aortic valve with thickened leaflets (arrows) and restricted opening consistent with aortic stenosis

Aortic stenosis (AS) is characterized by narrowing of the aortic valve, most commonly due to age related sclerosis, congenital bicuspid valve and rheumatic fever. It has a prevalence of 3- 5%. On MRI, the aortic leaflets are thickened and have low signal due to fibrosis/calcification. There is restricted opening of the valve leaflets in systole. The valve narrowing can be quantified by planimetry in dedicated short-axis views through the aortic valve (Fig. 4a). A dark dephasing jet is seen through the aortic valve in systole (Fig. 4b). The velocity of stenosis can be quantified using PC images obtained either perpendicular or parallel to the jet. The pressure gradient is derived by using the modified Bernoulli equation (ΔP = 4v^2^) and the stenosis is graded as shown in Table 4 [27]. The aortic valve morphology in AS is variable, and can be trileaflet, bicuspid, unicuspid (Fig. 4c) or quadricuspid, best evaluated in cine SSFP or gradient-echo images. Compensatory concentric LVH is seen in 52% of AS, which reduces the wall stress and maintains the cardiac output. Eventually, the left ventricle fails.

**Table 4:** Grades of aortic stenosis [27]

	Peak velocity (m/s)	Mean gradient	Valve area	Indexed valve area	Velocity ratio
Mild	2.6-2.9	<20^a^ (<30^b^) mm Hg	<1.5 cm2	0.85 cm2/m2	>0.5
Moderate	3.0-4.0	20-40^a^ (30–50 ^b^) mm Hg	1-1.5 cm2	0.60-0.85 cm2/m2	0.25-0.5
Severe	>4.0	>40^a^ (50^b^) mm Hg	<1 cm2	<0.6 cm2/m2	<0.25

^a^ AHA/ACC guidelines

^b^ ESC guidelines

Although concentric LVH is the most common type, asymmetric hypertrophy, concentric remodelling, asymmetric remodelling, normal geometry and decompensation are also seen in AS [22]. There is no correlation between the severity of valve narrowing and extent of LVH [22]. Asymmetrical thickening is seen in 22% of AS, involving the basal and mid septum, which makes this indistinguishable from HCM [22]. Longitudinal strain may be diminished in early stages. In late stages, the EF is diminished along with LV dilation. Wall stress and diminished perfusion is seen in later stages due to ischemia. LGE may be seen due to fibrosis, likely due to myocardial ischemia caused by higher demand of LVH and high systolic pressure, and decreased myocardial perfusion due to lower aortic pressure, lower diastolic duration and absence of coronary vasodilation [28, 29]. LGE is most commonly seen in the basal segments, typically in a diffuse subendocardial distribution, but occasionally in a mid-myocardial distribution [30, 31]. LGE is associated with severe hypertrophy and adverse prognosis. MRI is also useful in the evaluation of patients being considered for transcutaneous aortic valve implantation (TAVI), especially in patients with contraindication for CT, by measuring the annulus, sinus of Valsalva, annulus to coronary ostial distance, fluoroscopic angulation plane and also the dimension and orientation of the peripheral access vessels [32].

### Aortic coarctation

**Fig. 5:**
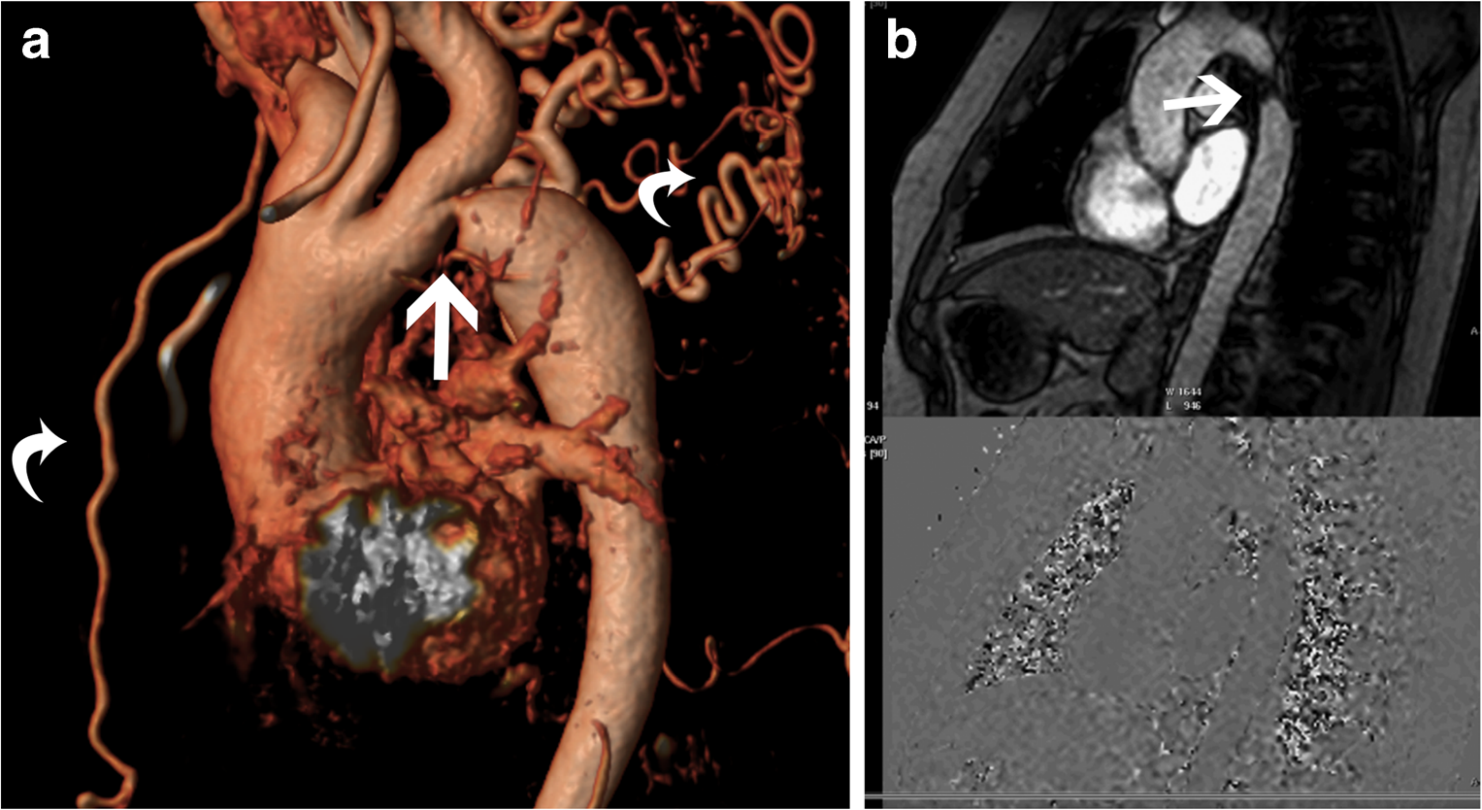
**a** Sagittal volume rendered 3D image in a patient with concentric LV thickening shows severe coarctation of the aortic arch (straight arrow) and multiple collateral vessels(curved arrows). **b** Velocity encoded phase contrast image in the sagittal plane in another patient shows flow acceleration through the aortic arch at the site of coarctation, which was quantified and the pressure gradient was estimated to be 20 mm Hg

Aortic coarctation is discrete narrowing of the aorta, usually seen beyond the level of ductus arteriosus, but may also be seen proximal to it. More extensive areas of involvement result in hypoplasia. Coarctation has a prevalence of 0.01 to 0.03% and is seen in 0.2% of hypertensive adult patients [33]. Coarctation produces concentric LVH due to pressure overload. Hypertension is seen either due to mechanical obstruction, activation of the renin-angiotensin system, neural reasons and/or Goldblatt type phenomenon [1]. On MRI, the coarctation and collateral vessels are evaluated and quantified using cine-SSFP or MR angiographic images (Fig. 5a). MRI can assess the presence and direction of flow in collaterals. The flow velocity, flow volume and pressure gradients at the coarctation site can be measured using a velocity-encoded PC sequence (Fig. 5b). Presence of higher flow in the distal segment of descending thoracic aorta compared to the proximal segment is an indicator of hemodynamically significant obstruction due to filling of the distal aorta by collateral vessels. LVH is seen in 20–40% of coarctation [33, 34]. Subendocardial ischemia and abnormal arterial stiffness are also seen [1]. Associations of coarctation include bicuspid aortic valve, ventricular septal defect (VSD), hypoplastic left heart and subaortic stenosis, all of which can be evaluated using MRI. MRI is used for preoperative/interventional planning and post-treatment assessment of complications.

### Subaortic membrane

**Fig. 6:**
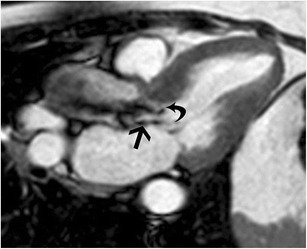
3-chamber cine SSFP image shows concentric LV thickening. There is flow acceleration in the LVOT below the level of the aortic valve (straight arrow). There is a linear discrete membrane at the LVOT (curved arrow), which is responsible for the flow acceleration, consistent with a subaortic membrane

The subaortic membrane is a discrete fibrous membrane, muscular narrowing or a combination of both. It usually involves the LV outflow tract (LVOT), but may also involve the septum, anterior mitral leaflet or the aortic valve. Common associations of subaortic membrane include bicuspid aortic valve, AV canal, Shone’s complex, tunnel subaortic stenosis and VSD. On MRI, focal discrete narrowing of the LVOT by a membrane is identified (Fig. 6) in cine SSFP and 3D whole heart SSFP images. Flow acceleration and dephasing is also identified (Fig. 6) in cine images, which can be quantified by using PC images. A peak gradient > 50 mm Hg is considered to be severe obstruction. Concentric LVH is present due to pressure overload and subendocardial ischemia may be seen. Complications include infective endocarditis and aortic regurgitation due to structural valvular damage. Treatment is with surgical resection when there is either hemodynamically significant obstruction or aortic valvular involvement [35].

## Volume overload

Volume overload conditions such as long-standing aortic or mitral regurgitation and dilated cardiomyopathy lead to complex forms of adaptation resulting in eccentric LV hypertrophy. The LVWT and LVWM are increased, but the RWM is lower than normal due to ventricular dilation. MRI can quantify the regurgitation as well as its secondary effects on the ventricle including abnormal chamber size and function. In dilated cardiomyopathy, the LV is dilated and there is global systolic dysfunction. A nonischemic pattern of LGE may be seen, in a mid-myocardial or subepicardial distribution depending on the underlying aetiology [36]. A linear mid-myocardial pattern has been described in idiopathic dilated cardiomyopathy [36].

## Infiltrative cardiomyopathies

Infiltrative cardiomyopathies are characterized by the deposition of abnormal substances in the myocardium resulting in diminished ventricular filling. Some of these entities cause LV thickening, whereas other entities predominantly manifest as chamber dilation and wall thinning but occasionally may manifest with wall thickening/remodelling. The former category includes cardiac amyloidosis, Fabry’s disease, Danon disease, eosinophilic heart disease, Friedreich ataxia, oxalosis and mucopolysaccaridoses, while the latter category includes sarcoidosis and iron overload cardiomyopathy [37].

### Cardiac amyloidosis

**Fig. 7:**
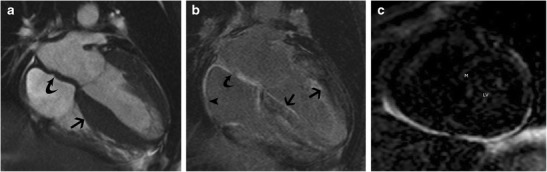
**a** 4-chamber SSFP image in a patient with cardiac amyloidosis shows concentric LV thickening (straight arrow). There is also thickening of the interatrial septum (curved arrow). **b** Post contrast T1-w inversion recovery gradient echo image shows diffuse sub-endocardial to mid myocardial LGE of the ventricles (straight arrow), interatrial septum (curved arrow) and the atrial walls (arrowhead). **c** Short-axis TI scout (Look Locker sequence image) at the level of mid-ventricular septum in a patient with cardiac amyloidosis. At an inversion time of 150 milliseconds, both the blood pool (LV) and myocardium (M) are dark. This is due to diffuse myocardial infiltration with amyloid, which takes up gadolinium and results in T1 shortening

Cardiac amyloidosis is characterized by the deposition of ß-pleated amyloid proteins, with an estimated prevalence of 0.001%. In the AL type, cardiac involvement is seen in 33%; in the AA type, cardiac involvement is unusual and in TTR-type; there are specific gene mutations [38]. On MRI, concentric LV thickening is seen, along with thickening of the RV, atria, interatrial septum and valves (Fig. 7a). Biatrial dilation and decreased LV volumes are also present. Longitudinal strain is decreased in the early stages and eventually EF is also decreased. There is no SAM or LVOT obstruction. Perfusion is usually normal, but a subendocardial defect may be seen. Due to interstitial expansion from amyloid deposition, LGE is seen in 69-97% of all cardiac amyloidosis patients, 100% of TTR type and 64% of AL type [38]. LGE is usually seen in a global transmural or subendocardial pattern (83%) (Fig. 7b), but occasionally may be focal and patchy (6%). In 8% of patients, a suboptimal myocardial nulling (8%) may be the only finding. LGE is seen in MRI in 47% of confirmed cardiac amyloidosis patients without echocardiographic findings [39]. LGE is associated with NYHA functional class, ECG voltage, LVM index, RV wall thickness, troponin T and BNP levels [37]. Alteration in T1 kinetics can be demonstrated in the T1-scout images, with nulling of the myocardium occurring at the same time or even before nulling of the blood pool (Fig. 7c) (which is the reverse of the normal pattern where the normal myocardium always nulls after the blood pool). At 2 minutes post contrast, a T1 difference between subepicardium and subendocardium of < 23 milliseconds (ms) is a predictor of mortality with 85% accuracy [38]. Native T1 mapping shows elevated T1 values, which is an early indicator and direct marker of cardiac amyloid [40]. Higher ECV has been shown with post contrast T1 mapping, which directly correlates with LGE, indexed LV mass and other clinical adverse prognostic factors [41].

### Anderson-Fabry’s disease

**Fig. 8:**
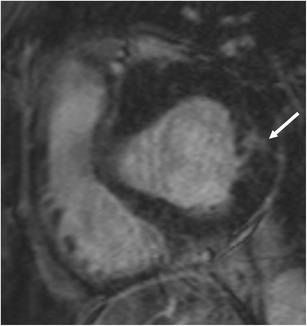
Short-axis post contrast T1-w inversion recovery gradient echo MRI sequence shows patchy mid myocardial LGE in the basal lateral (straight arrow) in a patient with Anderson Fabry’s disease

Anderson-Fabry’s disease is an X-linked storage disorder characterized by the accumulation of glycosphingolipid due to a deficiency of α- galactosidase enzyme. In the early stages, there is concentric LV thickening, prominent papillary muscles, preserved EF and diastolic dysfunction, which then progresses to intramural replacement fibrosis, regional wall motion abnormalities [42] and arrhythmias. On MRI, LV thickening is present, usually concentric but may be asymmetric. Fabry’s disease accounts for 3% of all LVH [43]. Fabry’s disease has been shown to be the aetiology in 6–12% of patients presenting with suspected symmetric HCM [44]. LGE is seen in up to 50% of patients with Fabry’s disease due to cardiac infiltration [45]. LGE in Fabry’s disease is typically seen in the basal inferolateral wall in a mid-myocardial or subepicardial pattern (Fig. 8). However, in HCM, LGE is seen in hypertrophied as well as non-hypertrophied segments, usually in a mid-myocardial distribution or at RV insertion points [45]. LGE is associated with poor prognosis. Native T1 values are low in Fabry’s disease due to presence of lipids, whereas in HCM it is high due to fibrosis. Elevated T2, RV involvement, valve thickening/dysfunction and decreased longitudinal systolic motion have also been reported in Fabry’s disease.

### Danon disease

**Fig. 9:**
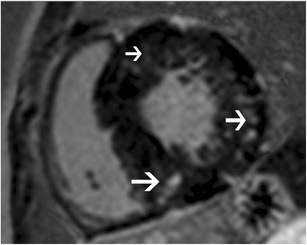
Short-axis post-contrast T1-w inversion recovery gradient echo image shows patchy mid myocardial LGE (arrows) of severely thickened myocardium in a patient with Danon disease

Danon disease is an X-linked dominant lysosomal storage disorder characterized by mutation of lysosomal associated protein-2 (LAMP2). Cardiac manifestation of Danon disease includes concentric LV thickening, cardiac failure and Wolff-Parkinson-White syndrome. Other features are myopathy, elevated creatine kinase, eye abnormalities and mental retardation. On MRI, concentric LV thickening is present. Danon disease has been confirmed as the correct diagnosis in 4% of patients initially diagnosed as HCM [46]. Myocardial oedema and perfusion defect are occasionally seen. LGE is seen in a non-ischemic pattern in a mid-myocardial distribution [47] (Fig. 9), but occasionally subendocardial and transmural distribution may be seen [48]. There is often sparing of the septum between the RV insertion points [49]. Involvement of the heart is associated with poor prognosis. Due to the risk of sudden cardiac death, implantable cardioverter defibrillator (ICD) placement or cardiac transplantation are performed.

### Eosinophilic endocarditis

**Fig. 10:**
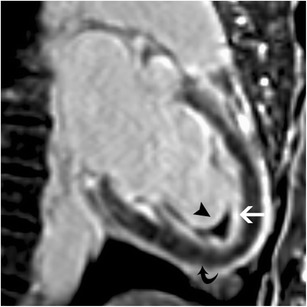
3-chamber post contrast T1-w inversion recovery image shows a triple layered pattern of enhancement with an inner layer of dark thrombus (arrowhead), middle layer of LGE due to enhancing fibrous tissue (straight arrow) and an outer layer of dark normal myocardium (curved arrow)

Eosinophilic endocarditis (endomyocardial fibrosis, Löeffler endomyocarditis) is a part of the spectrum of hyperesosinophilic syndrome, which is characterized by elevated eosinophils > 1500/ μL > 6 months and organ dysfunction. The heart is the most commonly involved organ and cardiac involvement is associated with adverse prognosis. There are three stages of cardiac involvement. In the acute necrotic stage, there is eosinophilic infiltration of the endocardium and myocardium. This is followed by the thrombotic-necrotic stage, which is associated with ventricular damage. In the late fibrotic stage, there is diffuse fibrous tissue lining the endocardium often extending from the LVOT to the apex. On MRI, there is thickening of the LV apex, which can be confused with apical HCM. A high T2 signal may be seen in the apical endocardium. Diffuse subendocardial perfusion defect may be present. On LGE, a triple layered pattern of enhancement is a characteristic finding of eosinophilic endocarditis. This includes an inner dark layer due to non-enhancing thrombus, a middle bright layer due to LGE from fibrous tissue and an outer dark layer of normally nulled myocardium (Fig. 10). Patchy intramyocardial LGE may also be present [50, 51]. Associated wall motion abnormalities and dilated left atrium may also be seen. Treatment is surgical resection of the fibrous tissue.

### Iron overload cardiomyopathy

**Fig. 11:**
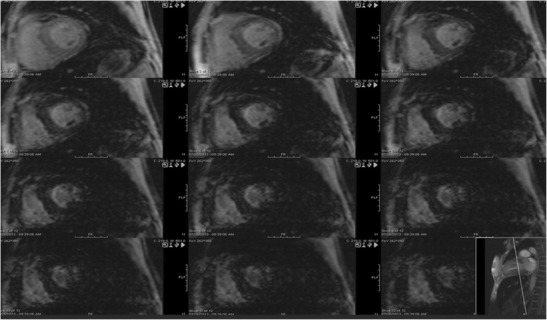
Short-axis multi-echo gradient echo T2 weighted images obtained at different TEs, shows that the myocardium gets progressively darker with increasing TEs, with complete darkness at TE of 14.2 milliseconds, indicating significant iron deposition

Iron overload cardiomyopathy is characterized by excess myocardial iron deposition, secondary to hemochromatosis, sickle cell disease, thalassemia, multiple transfusions or increased gastrointestinal (GI) absorption of iron [52]. It has an estimated prevalence of 0.0001%. There are two phenotypes of iron overload cardiomyopathy, the restrictive type with preserved EF, diastolic dysfunction, LA/RV dilation and pulmonary hypertension; and the dilated type with LV remodelling, chamber dilation and decreased EF [53]. Eccentric hypertrophy, high EF and high cardiac output may also be seen [53]. Iron overload cardiomyopathy can result in ventricular failure, arrhythmia and mortality [52]. Hence, it is the most important determinant of survival in patients with thalassemia or multiple transfusions. It is imperative to make the diagnosis of iron overload cardiomyopathy prior to the development of overt cardiac failure, since early initiation of chelation therapy can improve the outcome and reduce adverse events [52]. On MRI, multi-echo gradient echo images are obtained in the same slice at different TE values, and the mean myocardial signal intensity at each TE level is measured (Fig. 11). A curve fit is used to obtain myocardial T2. Normal myocardial T2* is 33 ± 6.5 milliseconds. Myocardial iron deposition is diagnosed when T2* is < 20 milliseconds and is considered severe, when T2* is < 10 milliseconds [54].

### Sarcoidosis

**Fig. 12:**
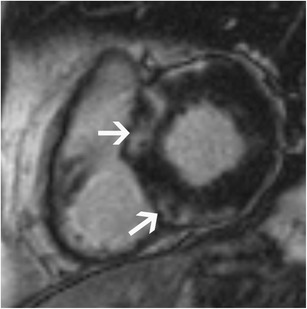
Short-axis post-contrast T1-w inversion recovery gradient echo image shows mild concentric LV thickening and patchy areas of mid myocardial and subepicardial LGE (arrows) in a patient with cardiac sarcoidosis

Sarcoidosis is a systemic disorder characterized by the presence of non-caseating granulomas. Cardiac involvement is seen in 5–20% of patients with sarcoidosis. In the acute stage, diffuse myocardial thickening may be seen due to diffuse infiltration. Regional wall motion abnormalities, myocardial oedema and LGE are also seen. LGE is seen in a mid-myocardial or subepicardial distribution. On STIR, confluent granulomas may be seen with a central low signal of fibrous tissue and a peripheral rim of high signal with a nodular component, more common in the basal septum than the lateral wall. In the chronic stage, patchy areas of mid-myocardial to subepicardial LGE may be seen with associated normal or thinned myocardium (Fig. 12) [55]. No oedema is seen in the chronic stage.

### Uncommon conditions

**Myocardial oxalosis** is characterized by the deposition of oxalic acid crystals in the heart due to its higher production. Clinical presentation includes LVH, heart block and conduction abnormalities. On MRI, there is concentric left and right ventricular thickening. Diastolic dysfunction may be seen. **Friedreich ataxia** is characterized by mitochondrial ion accumulation, with cardiomyopathy seen in 63%. On MRI, concentric or asymmetric LVH is seen, without evidence of LVOT obstruction. Diastolic dysfunction and fibrosis may be seen [37]. **Mucopolysaccharidoses** can present with variable MRI appearance depending on the phenotype, including asymmetrical septal thickening, mitral and or aortic valve stenosis/insufficiency with normal EF [37]. In **obesity**, LVH with high LVM, LVEDV and RWM is seen, with preserved EF [56] which correlates with the body mass. LVH with higher LVM with normal EF is also seen in **DM Type II**, correlating with BMI and HbA1c [57].

## Hypertrophic cardiomyopathy

**Fig. 13:**
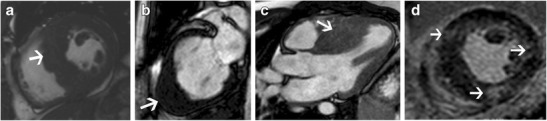
**a** Short-axis SSFP image shows severe asymmetric hypertrophy of the mid-ventricular septum, while the lateral, anterior and inferior segments appear to be of normal thickness. **b** 2-chamber SSFP image shows severe thickening of the apical LV segments, consistent with apical variant (Yamaguchi type) of hypertrophic cardiomyopathy. **c** 3-chamber cine SSFP image shows extensive mass-like appearance of a focal hypertrophic cardiomyopathy involving the mid-ventricular septum (arrow). **d** Short axis post contrast inversion recovery SSFP sequences shows patchy, fine mid-myocardial LGE of the hypertrophied septum, especially at RV insertion points (arrows) that are consistent with interstitial fibrosis in a pattern that is specific for hypertrophic cardiomyopathy

Hypertrophic cardiomyopathy (HCM) is characterized by myocardial hypertrophy without an underlying cause such as HT or AS. It is caused by sarcomeric gene mutations and has variable phenotypic expression. It has a prevalence of 0.2%, and LVH is seen in 60% of these patients [1]. The most common pattern of HCM is the asymmetrical hypertrophy, which typically involves the basal ventricular septum (Fig. 13a), but occasionally may involve the apical region (Yamaguchi type) (Fig. 13b), mid-ventricular region or even the lateral wall. Spiral and mass-like types (Fig. 13c) have also been described. The concentric type of HCM is rarely seen. On MRI, there is asymmetric hypertrophy, with a septal/lateral wall > 1. EF is either normal or hyperdynamic. Ventricular chamber size is normal but may be dilated in the end stages. In the obstructive type (HOCM), there is flow acceleration in the LVOT due to narrowing, which results in systolic dephasing. This can be quantified using velocity encoded PC imaging. SAM augments this obstruction and also results in eccentric posteriorly mitral regurgitation and left atrial enlargement. MRI can also help in evaluating papillary muscle abnormalities such as abnormal insertion, position and mobility which can augment the obstruction or may be the only cause of obstruction. Occasionally these abnormalities are seen without significant hypertrophy. While the classical HCM may need surgical myectomy or septal alcohol ablation, papillary muscle abnormalities require specific surgeries such as reimplantation of papillary muscles [58]. Mitral valve abnormalities, such as elongation, can also be seen in MRI [59], and these require mitral valve surgery. Stress perfusion defects may be seen. LGE is seen in 40–80% of HCM patients, either due to myocyte disarray, dysplasia of intramyocardial arterioles, interstitial fibrosis, or other types of fibrosis (plexiform, pericellular, perivascular, or substitution) [60]. LGE is typically patchy and seen in a mid-myocardial distribution, both in hypertrophied and non-hypertrophied areas, and also at RV insertion points (Fig. 13d). Detection and quantification of LGE is the most significant contribution of MRI in HCM, since the presence of LGE has been shown to be associated with adverse cardiovascular events including arrhythmias and sudden cardiac death, often warranting ICD placement [60–62]. LGE often underestimates the amount of fibrosis in HCM, since the fibrosis is diffuse, and; hence, establishing a normal myocardium to determine a threshold is challenging. T1 mapping is more sensitive in detection of diffuse fibrosis, with higher native T1 values and elevated ECV described in HCM. There is no correlation between LGE and T1 values, indicating that diffuse and regional fibrosis in HCM may be different entities [63].

## Athlete’s heart

Athlete’s heart is a physiological hypertrophy seen as an adaptive response to the altered loading conditions of prolonged and intensive training. This has been reported in an estimated 2% of athletes [64]. At a cellular level, the normal structural organization and function are preserved, and the signalling pathway for this response is also distinct (IGF-1/IGF-1R/Akt axis) from pathological hypertrophy [65]. The LV geometry is variable- concentric hypertrophy in strength-trained athletes due to pressure overload; eccentric hypertrophy in endurance-trained athletes due to volume overload; and a mixed pattern of concentric-eccentric hypertrophy in others.

**Fig. 14:**
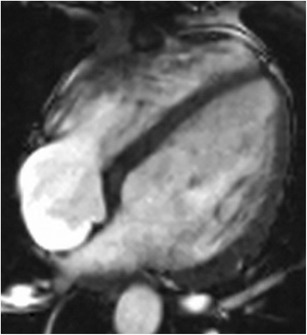
4-chamber cine SSFP image shows mild LV thickening (15 mm) and mild LV dilation (EDV-200 ml; EDVi- 120 ml/m2) with normal systolic function (EF- 59%). There was no abnormal LGE. This was diagnosed as athlete’s heart. After cessation of athletic training for three months, the ventricular measurements returned to normal

MRI shows hypertrophy, which may be concentric, eccentric or mixed concentric-eccentric (Fig. 14). Distinguishing athlete’s heart from HCM is critical, since missing a diagnosis of HCM puts the athlete at risk of sudden death and an erroneous diagnosis of HCM will disqualify an otherwise eligible athlete from contact sports [66]. Features that distinguish athlete’s heart from HCM are – the LVWT is < 15 mm in males and 13 mm in females; symmetrical thickening; normal septal/lateral wall ratio; dilated cavity (but end-diastolic diameter < 6.5 cm), resulting in low RWM of <1.16; diastolic wall-to-volume ratio < 0.15 mm x m2 x ml [66]; normal systolic function; normal/high diastolic function; and no LVOT gradient, SAM or perfusion defect. LGE is typically not seen unlike HCM; however, some studies have described LGE in12-50% of athletes [67, 68], with heterogeneous appearance, more commonly in the septum at the RV insertion site. Although the exact mechanism is unknown, theories include repetitive myocardial microtrauma, pulmonary artery pressure overload with dilated RV, genetic predisposition and silent myocarditis [68]. There may be proportional enlargement of the RV and atria. Clinical features that help in diagnosis are: absence of genetic abnormalities, milder ECG abnormalities (conduction delays, sinus bradycardia) and a peak oxygen uptake that is higher than expected for the age, gender and size. If the diagnosis of athlete’s heart still remains indeterminate after using all the abovementioned criteria, it may be confirmed by regression of the structural changes following a period of deconditioning.

## Neoplastic infiltration

**Fig. 15:**
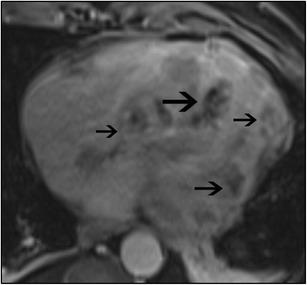
4-chamber post-contrast T1-w gradient echo first-pass perfusion image shows heterogeneous myocardial enhancement with areas of necrosis in a patient with metastatic deposits from a primary angiosarcoma of the extremity (arrows)

Diffuse LV thickening may also be seen due to diffuse or multifocal neoplastic infiltration. This includes neoplasms such as metastases, lymphoma, leukemia or sarcoma. B cell lymphoma is the most common type of lymphoma to produce diffuse myocardial infiltration [69]. Although cardiac leukemic infiltration is present in 37–44% of patients with leukemia, clinically it is seen only in 1% of these patients [70]. While leukemic deposits may not be separately discerned, lymphomatous and metastatic deposits are often grossly seen. On black-blood MRI, LV thickening is present, which is more irregular and has heterogeneous signal intensities compared to the other causes of LV thickening. The imaging appearance mimics HCM in non-contrast sequences, but in contrast-enhanced sequences, heterogeneous enhancement is noted (Fig. 15). Associated findings such as pericardial effusion may also be present.

## False positive hypertrophy

Occasionally, pseudohypertrophy may be seen due to partial averaging of normal structures. This may be seen due to a prominent moderator band, which may give a false impression of septal hypertrophy (Movies 1 and 2).

## Summary

MRI plays an important role in the comprehensive evaluation of patients with LV thickening. The unique tissue characterization capabilities of MRI enable establishing the aetiology of LV thickening, which is important for selecting the appropriate treatment. The high accuracy and reproducibility of MRI measured indices such as mass, volumes and function make it the ideal modality for serial follow-up in clinical scenarios and trials to evaluate treatment response. In addition, MRI also provides prognostic information.

## Electronic supplementary material

Below is the link to the electronic supplementary material.

Movie 1 Short axis cine SSFP sequence shows a focal area of thickening in the mid-portion of the ventricular septum. (AVI 147172 kb)

Movie 2 3-chamber cine SSFP sequence in the same patient shows that the abovementioned finding is indeed pseudohypertrophy due to a prominent moderator band. (AVI 147172 kb)
